# Zinc Oxide Nanostructures for NO_2_ Gas–Sensor Applications: A Review

**DOI:** 10.1007/s40820-014-0023-3

**Published:** 2014-12-16

**Authors:** Rajesh Kumar, O. Al-Dossary, Girish Kumar, Ahmad Umar

**Affiliations:** 1 PG Department of Chemistry, JCDAV College, Dasuya, 144 205 Punjab India; 2 Department of Physics, King Saud University, Riyadh, 11442 Kingdom of Saudi Arabia; 3 Department of Chemistry, Faculty of Arts and Sciences, Najran University, P.O. Box 1988, Najran, 11001 Kingdom of Saudi Arabia; 4 Promising Centre for Sensors and Electronic Devices (PCSED), Najran University, P.O. Box 1988, Najran, 11001 Kingdom of Saudi Arabia

**Keywords:** ZnO nanostructure, Gas sensors, Sensor parameters, Sensor mechanism

## Abstract

Because of the interesting and multifunctional properties, recently, ZnO nanostructures are considered as excellent material for fabrication of highly sensitive and selective gas sensors. Thus, ZnO nanomaterials are widely used to fabricate efficient gas sensors for the detection of various hazardous and toxic gases. The presented review article is focusing on the recent developments of NO_2_ gas sensors based on ZnO nanomaterials. The review presents the general introduction of some metal oxide nanomaterials for gas sensing application and finally focusing on the structure of ZnO and its gas sensing mechanisms. Basic gas sensing characteristics such as gas response, response time, recovery time, selectivity, detection limit, stability and recyclability, etc are also discussed in this article. Further, the utilization of various ZnO nanomaterials such as nanorods, nanowires, nano-micro flowers, quantum dots, thin films and nanosheets, etc for the fabrication of NO_2_ gas sensors are also presented. Moreover, various factors such as NO_2_ concentrations, annealing temperature, ZnO morphologies and particle sizes, relative humidity, operating temperatures which are affecting the NO_2_ gas sensing properties are discussed in this review. Finally, the review article is concluded and future directions are presented.

## Introduction

Tremendous increase in environmental pollution due to the fast industrialization, population growth, combustion of fuels from vehicles, use of pesticides and insecticides in agricultural sector, leakages of toxic chemical and gases is an alarming threat to ecosystems present in biosphere. The dissolution of harmful chemical from industrial effluent and runoff water from agricultural lands into running as well as underground water may result a number of health hazards. Early detection and monitoring of these poisonous and hazardous chemicals is thus required for environmental security purposes.

Chemical sensors play a significant role for the detection and monitoring of poisonous hazardous chemicals. Scientific community and researchers around the globe thus are trying to develop novel chemical sensors with superior performances. These chemical sensors also play other important and vital roles in other areas gas alarms, sensors for water and soil pollutants, human health, temperature sensor, speed sensor, magnetic field sensor, and emissions control [1, 2]. A sensor is a component of an electronic circuit which senses and undergoes physical and chemical changes on its surface due to the adsorption of a chemical stimulant. These adsorbed chemical species change the electrical properties of the sensor and subsequently convert these changes into measurable quantities [3–5].

Long ago in 1938, Wagner et al. observed remarkable change in the electrical properties at high temperatures for semiconductor materials on exposure to reducing or oxidizing gases [6]. Based upon these facts, Seiyama et al. for the first time developed semiconductor-based detector for gaseous components [7]. It was observed that at high temperatures of 400 °C, the adsorption and desorption of analyte gases results in a comprehensive change in electrical conductivity and resistivity of semiconductor materials.

From the variety of gas sensors, semiconducting metal oxide sensors such as ZnO [8–11], SnO_2_ [12–14], WO_3_ [15–17], CuO [18–20], Fe_2_O_3_ [21], In_2_O_3_ [22–25], CdO [26], TeO_2_ [27], and MoO_3_ [28] have been extensively used. Recently, ZnO nanostructures with variety of morphologies including nanorods [29–41]; nanowires [42–44]; nanofibres [45]; nanolines [46]; nanobelts [47]; nanoneedles [48]; nanoprism [49]; nanotubes [50]; nano/microflowers [51, 52]; quantum dots [54–56]; nanoparticles [57–60]; nanofilms, sheets, and plates [61–63]; nano/microspheres [64]; nanopyramids [65]; and nanotetrapods [66] have been extensively studied and applied for gas sensing applications as these materials under operating conditions possess high electron mobility, non-toxic nature, high-specific surface area, good chemical, and thermal stability [67, 68].

This review article presents recent developments for the synthesis and fabrication of ZnO, the n-type semiconductor-based gas sensors with variable morphologies, gas sensing mechanism, electrode assemblies, factors influencing gas sensing behavior of ZnO nanostructures, applications of ZnO-based nanomaterials as gas sensors for oxidizing gas analyte specially NO_2_ in this case and finally some drawbacks and limitations related to ZnO-based gas sensors.

## Chemical Behavior of NO_2_

NO_2_ is pungent red-brown oxidizing gas which enters the atmosphere from either natural sources or due to human activities. The latter are much more significant as they contribute reasonably a high NO_2_ concentration into the atmosphere. A large amount of oxides of nitrogen are disposed into the atmosphere due to fossil fuel combustion in automobiles and industries everyday. NO_2_ is regarded as a secondary pollutant produced from primary source NO which is being generated from internal combustion engines. In atmosphere, NO_2_ absorbs light in the ultraviolet region below 398 nm resulting in its photo-dissociation.

1 $${\text{NO}}_{2}+h\nu \left(<398\;\text{nm}\right)\to \text{NO}+\text{O}$$

These reactive species are converted into HNO_3_, organic, and inorganic nitrates including peroxyacetyl nitrate (PAN) through a series of chemical reaction (Eqs. 2–7).

2 $$\text{NO}\;+{\;\text{O}}_{3}\to {\text{NO}}_{2}+{\;\text{O}}_{2}$$

3 $${\text{NO}}_{2}+{\;\text{O}}_{3}\to {\text{NO}}_{3}+{\;\text{O}}_{2}$$

4 $$\text{NO}\;+{\;\text{NO}}_{3}\to 2{\text{NO}}_{2}$$

5 $$\text{O}\;+{\;\text{NO}}_{2}\to \text{NO}\;+{\text{O}}_{2}$$

6 $${\text{NO}}_{2}+{\;\text{NO}}_{3}\to {\text{N}}_{2}{\text{O}}_{5}$$

7 $${\text{N}}_{2}{\text{O}}_{5}+{\;\text{H}}_{2}\text{O}\to 2{\text{HNO}}_{3}$$

Hydrocarbons in the atmosphere produce peroxyacyl group which by an addition reaction of with NO_2_ produces PAN (Eq. 8).8

Prolonged exposure to NO_2_ can cause inflammation of lung tissue, bronchiolitis fibrosa obliterans, and silo-filler’s disease. Exposure of plants to several ppm of NO_2_ results in inadequate chlorophyll synthesis causing chlorosis and other plant tissue breakdowns. Still higher exposure can result in decreased rate of photosynthesis. Nitrogen dioxide also results in degradation of dyes and inks used in textile industries. NO_2_ emitted by supersonic jets in atmosphere causes the destruction of ozone layer present in the stratosphere which absorbs the harmful damaging UV radiation coming from the sun.

## Structure and Mechanism of ZnO for Gas Sensing Behavior

**Fig. 1:**
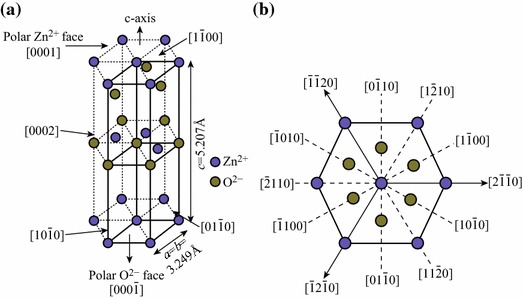
**a** ZnO unit cell with wurtzite structure. **b** Various crystal planes of ZnO Wurtzite structure

According to Yamazoe [69], there are two basic functions which a gas sensor consist of. These are receptor functions and transducer functions. Receptor function includes the recognition the chemical substance, whereas transducer function converts the chemical signal into electrical signals. This section deals with the structural properties favoring receptor functions of ZnO responsible for gas sensing behavior. ZnO has many different structural forms and shapes grown under different growth conditions. Wurtzite is the most favored form of ZnO at ambient conditions thermodynamically. The lattice constant parameters of wurtzite ZnO are *a* = 3.249 Å and *c* = 5.207 Å corresponding to *P63mc* space group with two interconnecting hexagonal-close-packed (hcp) sub-lattices in hexagonal lattice of Zn^2+^ and O^2−^ involving *sp*^3^ covalent bonding [70] (Fig. 1)

The ionic character of the material gives rise to a polar repeat unit along the *c*-axis. As a consequence of this polar symmetry, [0001] and $\left[000\overset{¯}{1}\right]$ surfaces of wurtzite ZnO exhibit different bulk terminations with the first one terminated by Zn-atoms and the latter by O-atoms. These are the most common crystal orientations of ZnO with different chemical and physical properties. As the repeating units of the crystal structure are perpendicular to the *c*-axis, a dipole moment exhibited the Madelung energy diverges at these surfaces for an ideal bulk truncation. This is a general property of ‘polar surfaces’ and consequently such bulk-truncated surfaces cannot be stable. Despite this inherent instability, the polar [0001] and the $\left[000\overset{¯}{1}\right]$ surfaces are among the most common crystal orientations of ZnO. To stabilize these polar surfaces, additional positive or negative charges are required. This implies that there exist efficient stabilization mechanisms of these surfaces that allow a convergence of the Madelung energy (electrostatic potential). These stabilization mechanisms may also influence the gas sensing properties of ZnO [71–76]. ZnO is an n-type semiconductor with electrons as current carriers. The adsorption of molecular and atomic oxygen on the surface of ZnO nanoparticles creates an electron-depleted space-charge layer, an important characteristic of the receptor function. This adsorbed oxygen further determines the surface charge layer thickness, surface potential barrier height, surface charge, and Debye length [77–79]. These parameters strongly affect the gas response and the selectivity of the ZnO gas sensors. The easy hydroxylation of the ZnO [0001] causes a metallization of the surface which can affect the conductivity response of such samples. The studies of the surface properties of the polar surfaces indicate that the differences in the chemical properties of the two polar surfaces affect the chemisorption of molecules and this plays an important role in gas sensing of such systems.

Han et al. [80] demonstrated that the gas sensing performance of ZnO is affected by its crystal defects. Greater the extent of oxygen vacancies higher is the gas response of ZnO [81]. In addition to this, the gas sensing performance of ZnO is also greatly found to be dependent on the size and morphology [82].

**Fig. 2:**
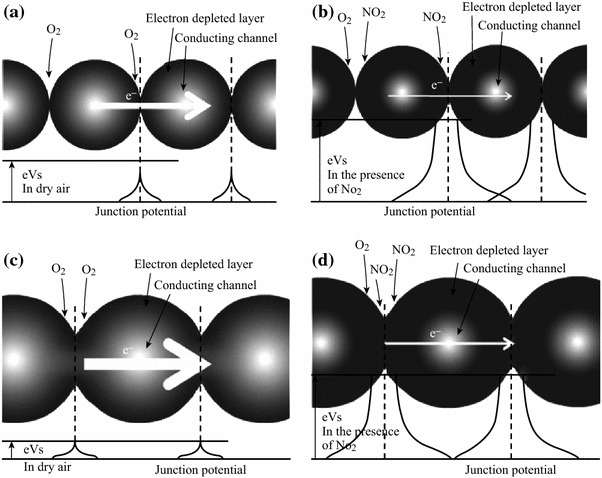
Schematic diagrams representing potential of **a**, **b** ZnO nanoparticles with grain-boundary junctions in the presence of dry air and NO_2_ gas, respectively. **c**, **d** ZnO nanoparticles with necked junctions in the presence of dry air and NO_2_ gas, respectively. Thickness of the *arrows* is related to the amount of electron current carriers. Reprinted with permission from [59]. © 2009 Elsevier B.V

The second function, viz. transducer function, depends upon interactions of analyte gas and the ZnO nanoparticles. Two types of interactions are considered to be important which may be either grain-boundary or neck interactions. As far as the grain-boundary contacts are considered, the movement of the electrons occurs for each boundary across the surface potential barrier. As a result, the barrier height is altered and consequently the electric resistance of the sensor material is also changed [83]. However, the resistance and the gas response are independent of the particle size (Fig. 2a, b).

**Fig. 3:**
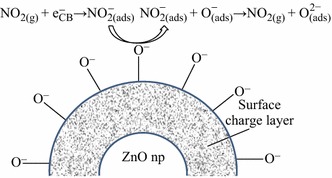
Schematic representation of receptor functions of ZnO gas sensor

On the other hand for neck contacts, the transfer of the electron occurs through channels formed at each neck as a result of the space-charge layer. This channel width is controlled by the neck size; any change in the width alters the material resistance [59, 84] (Fig. 2c, d). With decreasing particle size, the sensor material is devoid of the mobile charge carriers and thus only the space-charge layer region dominates. The energy bands become flat throughout the interconnected particles. As a result, there is no significant barrier for intercrystallite charge transport. However, high-temperature sintering of the ZnO nanomaterials for making porous layers on different substrates results in agglomeration, thereby increasing grain size. Adsorbed NO_2_ gas on the surface of the ZnO nanoparticles gets reduced by the transfer of the electrons from the conduction band (Fig. 3) of ZnO, thereby broadening the electron-depleted layer and hence resulting in wider junction potential barriers as shown in Fig. 2. This increases the resistance of the ZnO layer with widening of the junction potential barriers. This forms the basis of applications of ZnO nanomaterials as potential gas sensors. Exceptionally, Wang et al. [50] observed a p-type semiconductor behavior for ZnO nanotubes at low operating temperature of 30–50 °C which switches over to n-type behavior at high temperature for NO_2_ concentrations ranging between 500 ppb and 10 ppm.

## Basic Gas Sensing Characteristics

In the literature, a number of gas sensing parameters such as gas response (*R*), response time (*τ*_res_), recovery time (*τ*_rec_), analyte concentration, operating temperature, and detection limit for ZnO-based gas sensors are reported. These parameters are elaborated in this section.

### Gas Response

Gas response of a gas sensor is generally defined as the ratio of the resistance change on the surface of the gas sensor before and after being exposed to the gas analyte. It is mathematically expressed in different forms by different group of researchers as shown in Eqs. 9 [35–38, 40, 41, 43, 47, 49, 50, 52, 54–62, 64, 65], 10 [29, 34, 39, 42, 44, 66], 11 [31, 48, 60, 63] and 12 [30, 53].

9 $$R=\frac{R_{\text{g}}}{R_{\text{a}}}$$

10 $$R=\frac{R_{\text{g}}-R_{\text{a}}}{R_{\text{a}}}=\frac{\Delta R}{R_{\text{a}}}$$

11 $$R=\frac{R_{\text{g}}-R_{\text{a}}}{R_{\text{a}}}\times 100=\frac{\Delta R}{R_{\text{a}}}\times 100$$

12 $$R=\frac{I_{0}-I_{\text{g}}}{I_{\text{g}}}=\frac{\Delta I}{I_{\text{g}}},$$

where *R*_a_ is the sensor resistance in presence of ambient dry air and *R*_g_ the sensor resistance in the presence of target gas; *I*_0_ the reference value (baseline) of the ZnO nanomaterials in dry air ambient and *I*_g_ the current of the ZnO nanomaterials in the presence of target gas.

### Response Time

Response time, is the time taken by a gas sensor upon exposure to a target gas from the first reaction to the stable end value when the signal has reached a particular percentage level (in general taken as 90 % in many reports and is usually represented as *T*_90_) [13–36, 38–45]. Further, lower the response time better are the sensing properties of the sensor. The response time is short for higher concentrations of target gases. Therefore, care should be taken for monitoring toxic gases at low concentrations as they may take a longer response time. In ZnO-based gas sensor, typical response time may be of the order of a minute or less. Other parameters which may affect the response time are NO_2_ flow rate, temperature, and pressure of the analyte gas.

### Recovery Time

Recovery time is the time required by a sensor so as to return to 90 % of the original baseline signal when the target gas is removed and the sensor is subsequently cleaned with dry air [29–34]. For good sensor applications, sensor recovery time should be small so that the sensor can be used over and over again.

### Selectivity

In general, the selectivity of a gas sensor material means the preferential chemiresistive sensing for a particular gas in the presence of another gas under similar operating conditions. The selectivity of ZnO-based nanosensors can be expressed as in Eq. 13 [63].

13 $$\text{Selectivity}=\frac{\text{Sensitivity of the sensor toward interface gas}\;(S_{\text{i}})}{\text{Sensitivity of the sensor toward target gas}\;(S_{\text{g}})}$$

The ZnO film used as sensor by Chougule et al. [63] showed high selectivity for NO_2_ over H_2_S compared to NH_3_.

### Detection Limit

For high-performance sensor applications, sensor should be capable to detect even very low concentrations of the gases. The minimum concentration of analyte gas which can be detected by a sensor under operative conditions is called its lower detection limit [29–31]. For ZnO nanorod-based gas sensors, the lowest detection limit of 50 ppb at 300 °C [29], 10 ppb at 250 °C [30], and 100 ppb at 250 °C [31] has been reported for NO_2_ gas.

### Stability and Recyclability

Stability of the sensor material refers to its ability to maintain its sensing properties repeatedly and even for long durations. ZnO sensor exhibits relatively high response which drops with time due to interface modification during operation to a steady state [63].

All these parameters depend on the nature, particle size and morphology of the sensing material, the type of interactions and reactions occurring between the gas and the sensor material, the sensor operating conditions, etc. In order to control these parameters, scientific understanding of gas–sensor interaction and various new technological concepts and novel materials have been developed, and some of these issues will be discussed in the latter sections.

## Zinc Oxide Nanostructure-Based NO_2_ Gas Sensors: Synthesis, Growth, and Characterization

Intense research efforts to synthesize ZnO nanomaterials and to fabricate efficient miniaturized devices using these materials for the applications in various nano-electronics and nanosensors are on a go around the globe. For this, a variety of fabrication techniques and methods have been explored in the literature for the synthesis of zinc oxide nanostructures. Varieties of morphologies for ZnO have been synthesized using various physical and chemical methods explained in the next section.

### 1-D ZnO Nanomaterials (Nanorods, Nanowires, Nanotubes, and Nanofibres)

The 1-D ZnO nanostructures, for instance nanorods, nanotubes, nanowires, and nanofibres, have been extensively studied for gas sensing applications due to their high surface-volume ratio, crystallinity, and charge confinement ability. A number of methods are reported in literature for the fabrication of 1-D ZnO nanostructures for gas sensors. Quantity and quality of 1-D ZnO nanostructures, however, vary widely from process to process. These synthetic processes can be broadly classified into two categories like (a) wet processing routes including hydrothermal methods, sonochemical growths, chemical bath depositions, etc. These methods may or may not involve the use of capping agents, (b) vapor-phase processing routes which include various sputtering techniques, thermal evaporation, and vapor-phase transport. For the growth of 1-D ZnO nanostructures, processing details are summarized in Table 1.

**Table 1:** 1D-ZnO nanostructures: morphology, methods of preparation, size, and other growth parameters

Morphology	Synthetic method	Growth reagents	Substrate	Anne. temp. (°C)	Anne. time (in h)	Particle size		Ref.
						Diameter (nm)	Length (nm)	
Nanorods	Hydrothermal method	Zn(NO_3_)_2_·6H_2_O, (HMTA)	Glass	500	2	30–120	250	[29]
Nanorods	Hydrothermal method	Zn(NO_3_)_2_·6H_2_O, HMTA	Alumina	–	–	100	1.62 μm	[30]
Nanorods	Sonochemical method	Zn(NO_3_)_2_·6H_2_O, HMTA	ITO coated glass	–	–	50	500	[31]
Nanorods	Hydrothermal method (different growth temperatures)	Zn(CH_3_COO)_2_·2H_2_O, diaminopropane, (HMTA)	Alumina	–	–	33	1.00 μm (90 °C) 1.43 μm (100 °C) 1.78 μm (110 °C)	[32]
Nanorods Nanosheets	Electrochemical deposition	ZnCl_2_, KCl	Porous silicon	–	–	200	700	[33]
Nanorods	Hydrothermal method	–	–	–	–	10	Several tens of nm length	[34]
Nanorods Nanoprisms	Continuous hydrothermal flow synthesis	Zn(NO_3_)_2_·6H_2_O, KOH	–	–	–	–	–	[35]
Nanorods with nanovoids	Hydrothermal method	ZnCl_2_, P123	–	500	8	23	900	[36]
Au/ZnO nanorods ZnO nanorods	Hydrothermal method	Zn(NO_3_)_2_·6H_2_O, CTAB	–	600	1	100–150	1–1.5 μm,	[37]
Hierarchical nanoclusters were built from 1-D nanorods	Chemical bath deposition	Zn(CH_3_COO)_2_·2H_2_O, ethaholamine	Quartz	500	2	1 μm	2.5 μm	[38]
Ce/ZnO nanorods	Hydrothermal method	Zn(NO_3_)_2_·6H_2_O, Ce(NO_3_)_3_·6H_2_O, (HMTA)	Al_2_O_3_	300	1	–	915–1,915 nm	[39]
Needle-like nanorods	Reverse microemulsion	Zn(CH_3_COO)_2_, SDS	–	400, 600, 800	4	52	3 μm	[40]
Pencil-like nanorods	CTAB-assisted hydrothermal	Zn(NO_3_)_2_·6H_2_O, CTAB		400, 600, 800	4	90	4 μm	
Flower-like nanorods	PEG-assisted hydrothermal	Zn(CH_3_COO)_2_, PEG		600	4	52	3 μm	
Pencil-like nanorods	CTAB-assisted hydrothermal	Zn(NO_3_)_2_·6H_2_O, CTAB	–	–	–	20	3–5 μm	[41]
Unbranched nanowires Branched* nanowires	Vapor-phase growth	ZnO/C	Si sapphire	–	–	Mean diameter of few nm	Lengths of few μm	[42]
Nanowires	Carbothermal reduction	ZnO/C	SiO_2_/Si	–	–	80–120	10 μm	[43]
Functionalized nanowires	Vapor-phase growth	Pure Zn	Al_2_O_3_	–	–	50	5–10 μm	[44]
Nanobarbed fibers	Electrospinning followed by chemical bath deposition	Zn(CH_3_COO)_2_·2H_2_O, HMTA	–	–	–	140–210	175–850	[45]
Nanolines	Sol–gel method	Zn(CH_3_COO)_2_·2H_2_O, 2-methoxyethanol and ethanol amine	–	700	1	Nanoline gap between 100 and 400 nm	–	[46]
Nanobelt	RF sputtering	ZnO	Sapphire	–	–	10	50	[47]
Nanoneedles Cacti-like structure	Low-temperature hydrothermal process	Zn(CH_3_COO)_2_·2H_2_O, diaminopropane, HMTA	Glass	–	–	15	300	[48]
Nanoprisms	Sol–gel method	Zn(CH_3_COO)_2_·2H_2_O, poly vinyl alcohol	Al_2_O_3_	700	–	25	100–500	[49]
Nanofibers	Electrospinning methods			400, 700				
Nanotubes	Hydrothermal method	ZnCl_2_, methenamine	Silicon (100) wafer with a thin SiO_2_	400	6	200	–	[50]

**Fig. 4:**
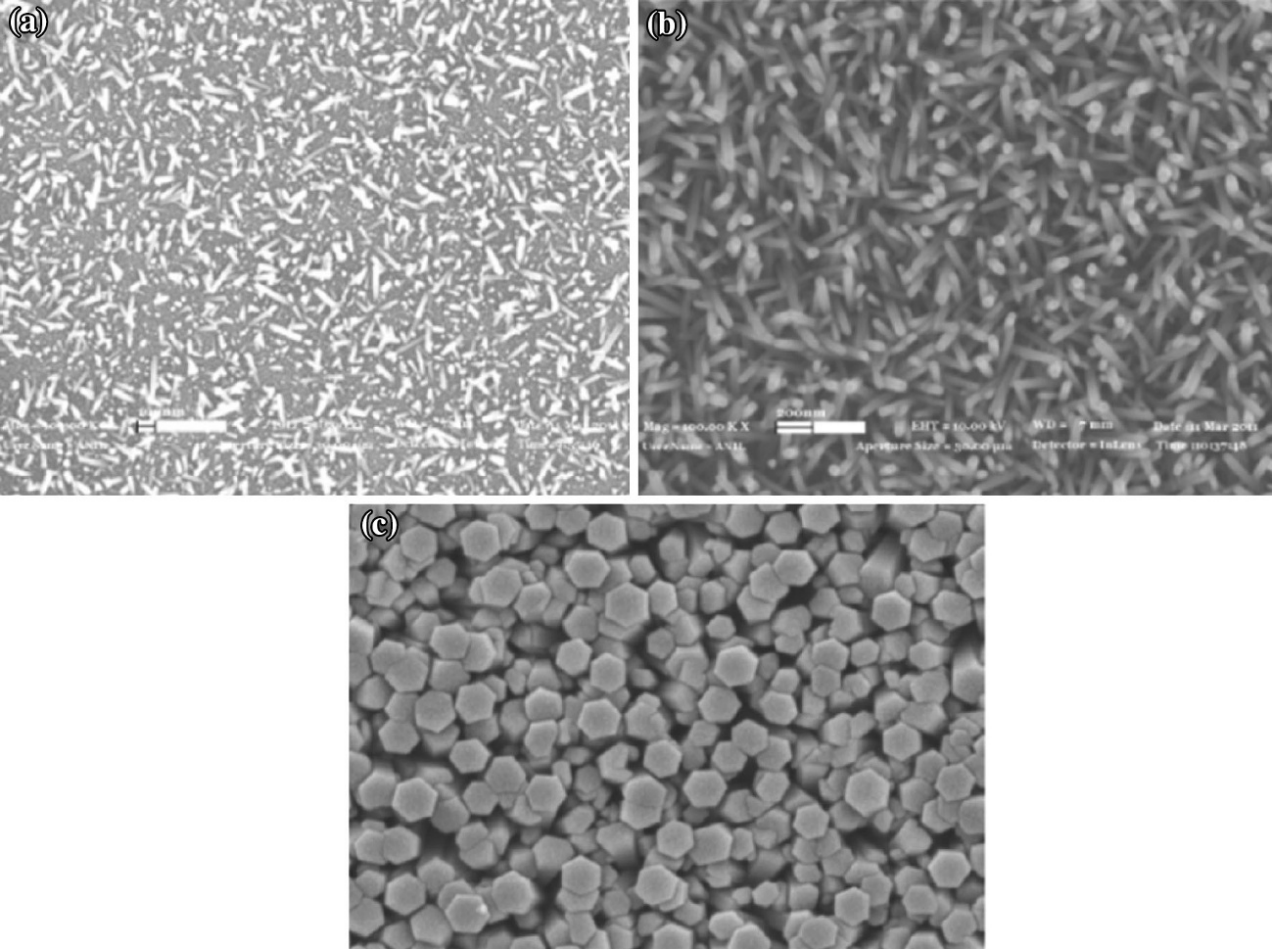
SEM images of ZnO nanorods for equimolar zinc nitrate and HMTA solution, **a** 0.001 M, **b** 0.01 M, **c** 0.1 M. Reprinted with permission from [29]. © 2013 Elsevier B.V

Ozturk et al. [29] fabricated ZnO nanorods by hydrothermal method using Zn(NO_3_)_2_·6H_2_O and hexamethylenetetramine (HMTA) precursors in equimolar ratios on seed layer. Seed layers were grown on glass substrate by sol–gel deposition method. Product obtained was annealed in air at 500 °C for 2 h. The dimensions of the as-synthesized nanorods were controlled by the different concentration of the precursors. With increasing concentration, the diameter was increased, whereas the length of the nanorods was almost constant (Fig. 4). It was observed that if equimolar mixtures of ZnO and HMTA were not used, no formation of ZnO nanorods was observed.

Increase in diameter can be attributed to wurtzite hexagonal crystal structures of ZnO with active polar faces [0001], $\left[000\overset{¯}{1}\right]$ and stable non-polar faces $\left[01\overset{¯}{1}0\right]$, $\left[0\overset{¯}{1}10\right]$, $\left[1\overset{¯}{1}00\right]$, $\left[\overset{¯}{1}100\right]$$\left[\overset{¯}{1}010\right]$, $\left[10\overset{¯}{1}0\right]$. The latter is responsible for the radial growth of ZnO nanorods (Fig. 1).

**Fig. 5:**
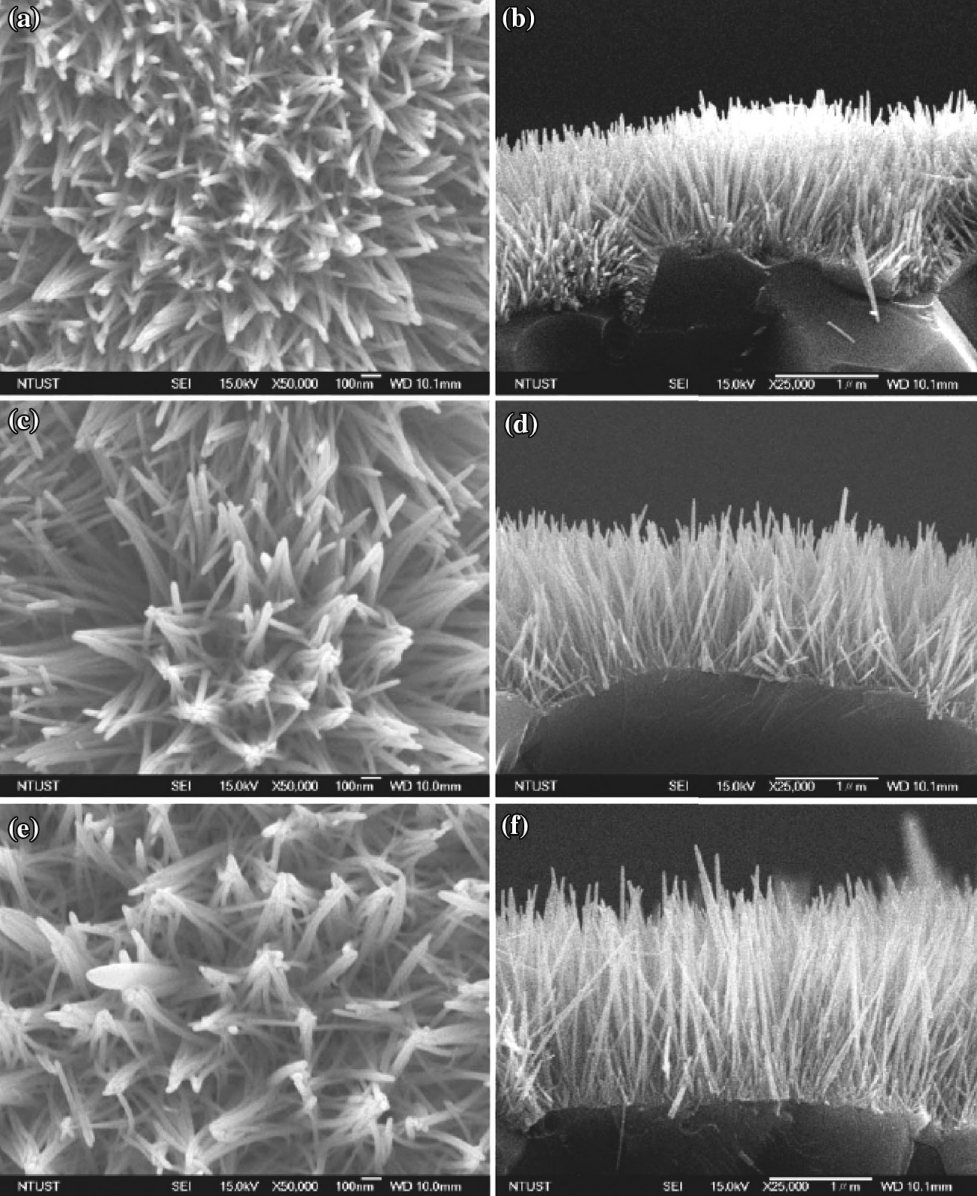
SEM micrographs of ZnO nanorod grown at **a** 90 °C, top view; **b** 90 °C, side view; **c** 100 °C, top view; **d** 100 °C, side view; **e** 110 °C, top view; **f** 110 °C, side view. Reprinted with permission from [32]. © 2009 Taiwan Institute of Chemical Engineers published by Elsevier B.V

Sahin et al. [30] used Zn(NO_3_)_2_·6H_2_O and HMTA precursors for the synthesis of unannealed ZnO nanorods of diameter 100 nm and the length was 1.62 µm from ZnO thin film seed layer coated with indium tin oxide (ITO) glass substrate by hydrothermal method. Using same precursors, Oh et al. [31] sonochemical grew high-density ZnO nanorod arrays on Pt-electrode patterned alumina substrate. Liu et al. [32] prepared ZnO nanorods of 30 nm diameter and 1.78 µm length for seeded growth using equal molar ratio of zinc acetate dehydrate and HMTA on sputtered platinum interdigitated circuits over alumina plates as electrodes. The length of the nanorods was found to increase from 1.00 to 1.78 µm as the growth temperature was increased from 90 to 110 °C, whereas the diameter on the nanorods was found 33 nm for each growth temperature (Fig. 5). The observation is quite different from the effect of concentration of the precursors which results in radial growth increasing diameter without affecting the length of the nanorods. From this, it can be concluded that concentration of the precursors controls the radial growth along non-polar faces$\left[01\overset{¯}{1}0\right]$, $\left[0\overset{¯}{1}10\right]$, $\left[1\overset{¯}{1}00\right]$, $\left[\overset{¯}{1}100\right]$$\left[\overset{¯}{1}010\right]$, $\left[10\overset{¯}{1}0\right]$, while temperature has marked influence on active polar faces [0001], $\left[000\overset{¯}{1}\right]$ of the hexagonal wurtzite crystal structures of ZnO (Fig. 1).

In addition to the concentration and the temperature, the morphology and size of the ZnO nanomaterials is also affected by the pH of the solution. In this direction, Yan et al. [33] studied the effect of pH for electrochemically deposited ZnO at 65 °C using ZnCl_2_ and KCl and a three-electrode glass electrochemical cell constituting Pt working electrode, counter electrode, and SCE electrode as reference electrode. At electrolyte pH = 6, dense zinc oxide nanosheets were formed on the porous silicon wafer substrate. When the pH of the electrolyte was increased to 6.5, ZnO nanosheets and nanorods arrays were formed on the surface of the porous silicon. Dendritic structures of ZnO were formed at still higher pH of 7. Thus, the presence of OH^−^ left in the solution plays a key role in the ZnO morphology. It must be due to the fact that ZnO is a polar crystal having positive polar [0001] plane comprising Zn^2+^ cations and a negative polar $\left[000\overset{¯}{1}\right]$ plane rich in O^2−^ anions. Anionic [Zn(OH)_4_]^2−^ complex formed as a result of reaction between Zn^2+^ and OH^−^ is preferably adsorbed on the positive polar [0001] plane of hexagonal ZnO nuclei. This leads to intrinsic anisotropy growth along the *c*-axis direction of the ZnO (Fig. 1) thereby resulting 1D nanostructures of ZnO such as nanorods formation at higher pH.

**Fig. 6:**
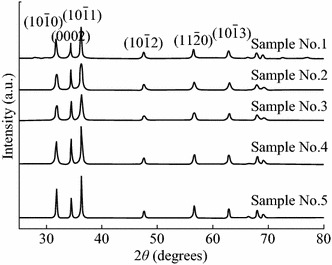
XRD patterns of ZnO nanopowder with 0, 0.05, 0.1, 0.15, and 0.25 [H_2_O_2_] to [Zn] ratios, respectively. Reprinted with permission from [35]. © 2013 American Chemical Society

**Fig. 7:**
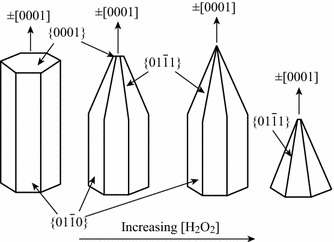
Proposed growth directions for ZnO rods and pyramids with increasing [H_2_O_2_] to [Zn] ratios, respectively. Reprinted with permission from [35]. © 2013 American Chemical Society

Shi et al. [35] studied the effect of volume ratios of H_2_O_2_ to zinc nitrate hexahydrate as precursors. The presence of H_2_O_2_ promotes faster growth along the [0001] direction by increasing the solubility of Zn^2+^ species in solution. At higher [H_2_O_2_] to [Zn] ratio, the tapering of one end of the ZnO nanorods along the [0001] direction may be attributed to the suppression of the [0002] plane. The fact is supported by the XRD patterns for the ZnO samples formed (Fig. 6). At still higher [H_2_O_2_] to [Zn] ratios, a tapered nanopyramid-like structures with sharp [0001] basal plane and a $\left[01\overset{¯}{1}1\right]$ radial plane are formed (Fig. 7).

Han et al. [36] synthesized single-crystal ZnO nanorods with nanovoids via a simple hydrothermal method for fabricating NO_2_ gas nanosensors.

**Fig. 8:**
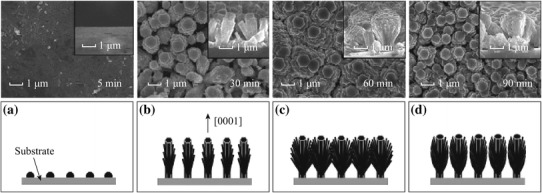
The SEM images and growth mechanism for ZnO nanostructures films deposited for various deposition time (5, 30, 60, and 90 min). Reprinted with permission from [38]. © 2012 Elsevier B.V

Deposition time is yet another key factor in determining the morphology of the nanostructures. Figure 8 represents the schematic representation of the growth mechanism for 1-D ZnO nanomaterials grown by Xu et al. [38] from ZnO thin film on quartz substrates.

**Fig. 9:**
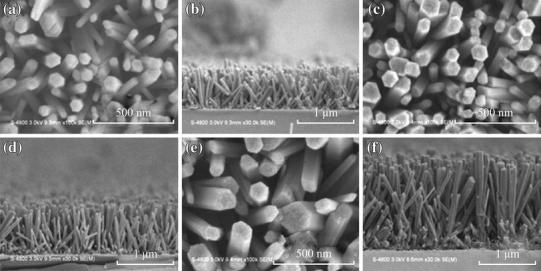
Top-view FESEM images of Ce/ZnO nanorods at different growth times. **a** 2 h, **c** 4 h and **e** 24 h and cross-sectional FESEM images at different growth times. **b** 2 h, **d** 4 h and **f** 24 h. Reprinted with permission from [39]. © 2014 Elsevier Ltd and Techna Group S.r.l

It can be observed that with increase in deposition time the growth of ZnO nanorods along the [0001] direction, perpendicular to hexagonal basal planes, is enhanced. A lower concentration of Zn^2+^ from a critical value results in no nucleation and as a result, the size of nanoclusters formed is smaller. Chang et al. [39] compare the effect of growth time on the structure and morphology of Ce-doped ZnO nanorods. Increasing the growth time from 2 to 4 h and then to 24 h, the lengths of the highly uniform hexagonal ZnO nanorods were 915, 955, and 1,915 nm, respectively with the diameter range from 97nm to137 nm (Fig. 9). This may be attributed to the growth along non-polar faces $\left[01\overset{¯}{1}0\right]$, $\left[0\overset{¯}{1}10\right]$, $\left[1\overset{¯}{1}00\right]$, $\left[\overset{¯}{1}100\right]$$\left[\overset{¯}{1}010\right]$, $\left[10\overset{¯}{1}0\right]$ as well as along the active polar faces [0001], $\left[000\overset{¯}{1}\right]$ of the hexagonal wurtzite crystal structures of ZnO with growth time [85].

The morphology and dimensions of ZnO nanorods can also be controlled by the type of additives, their nature and base molar ratio (OH^−^:Zn^2+^), etc. Pencil-like ZnO nanorods and needle-like ZnO nanorods were synthesized by the cetyltrimethyl ammonium bromide (CTAB)-assisted hydrothermal process at low temperature and via reserve microemulsion method, respectively, whereas the flower-like ZnO nanorods were fabricated by the polyethylene glycol (PEG)-assisted hydrothermal process by Bai et al. [40, 41]. Ionic surfactant like CTAB directs the growth and prevents the agglomeration for the anisotropic growth of 1D crystalline nanorods, with high crystallinity and high aspect ratio. [Zn(OH)_4_]^2−^ growth units generated for ZnO crystal during hydrothermal process are believed to be transported by CTAB to the polar [0001] plane resulting in the elongation of ZnO nanorods [41]. The optimized base molar ratio (OH^−^:Zn^2+^) of 6:1 was found to be appropriate for high-quality ZnO nanorods growth.

**Fig. 10:**
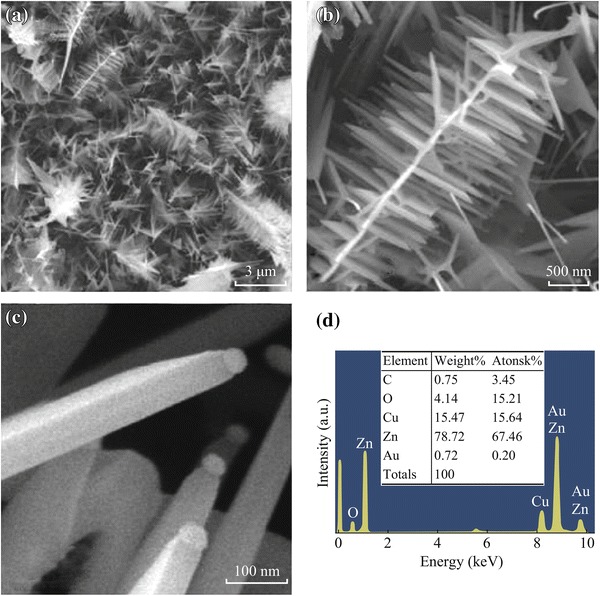
**a**, **b** Low- and high-magnification SEM image of branched ZnO nanowires. **c** Very high-magnification SEM image of globular Au particle at the tip of each secondary branch of a typical branched ZnO nanowire. **d** EDX spectrum of nanograined ZnO nanowires. Reprinted with permission from [42]. © 2013 Elsevier B.V

1-D ZnO nanowires have also been reported in the literature as efficient chemical and biological sensors. Seeded growth approach was adopted by An et al. [42] for the fabrication of branched ZnO nanowires in a three-step process. Unbranched ZnO nanowires initially grown on C-plane sapphire substrates were coated with a thin film of Au catalyst by direct current (DC) magnetron sputtering followed by thermal evaporation process. Figure 10a and b presents FESEM image of the as-synthesized branched ZnO 1-D dendrite-like nanowires having branches with diameter of few tens of nanometers and lengths of a few micrometers at low and high resolution, respectively, whereas Fig. 10c exhibits the morphology of the ZnO nanowires with globular Au particle which act as catalyst for secondary branch of a typical branched ZnO nanowire. Figure 10d reveals EDXS of the nanowires with an atomic weight ratio of O and Zn of 4:4. This indicates that the branched ZnO nanowires are highly rich in Zn contents.

Ahn et al. [43] fabricated ZnO nanowire for NO_2_ gas sensing on patterned Au catalysts by a selective growth of nanowires using ZnO/C mixture. In another vapor-phase growth, Waclawik et al. [44] carried out uncatalyzed ZnO nanowire growth on alumina substrates using pure Zn powder as precursor. As grown ZnO Nanowires were further functionalized by tris(hydroxymethyl)aminomethane (THMA) and dodecanethiol (DT) for NO_2_ gas detection. To study the NO_2_ sensing behavior, Lee et al. [45] utilized electrospinning and chemical bath deposition methods for the synthesis of ZnO nanobarbed fibers by the epitaxial growth of 1-D ZnO nanorods on ZnO nanofibers. ZnO nanostructures with other variety of morphologies including nanolines [46] nanobelts [47], nanoneedles [48], and nanotubes [50] have also been reported in the literature and applied for NO_2_ gas sensing.

### ZnO Nano/Microflowers

**Fig. 11:**
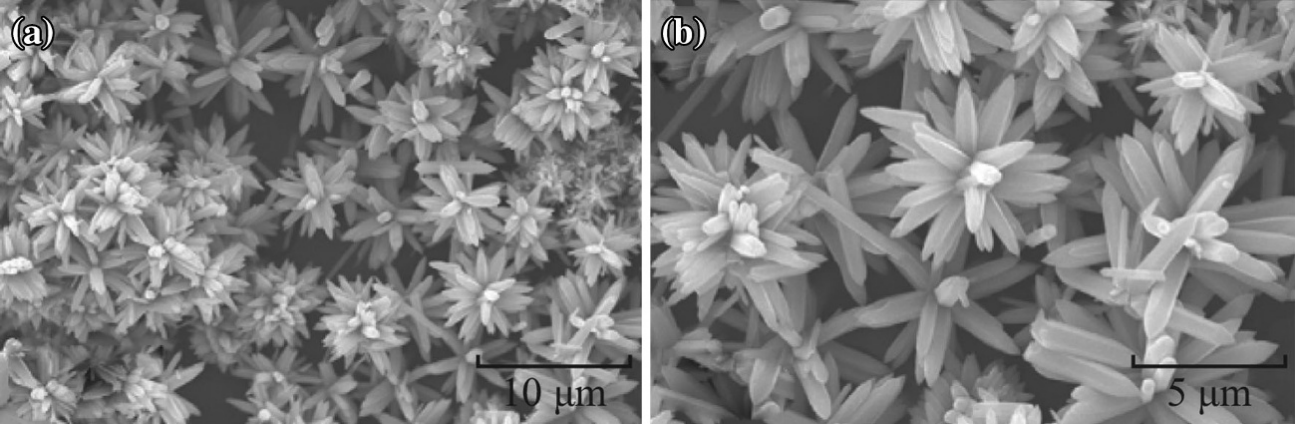
FESEM images of flower-like ZnO microstructure; **a** low magnification, **b** high magnification. Reprinted with permission from [51]. © 2013 Elsevier B.V

There are many other ZnO nanostructures with different morphologies other than 1-D nanomaterials which can be efficiently explored for gas sensing properties (Table 2). Rai et al. [51] in their two step growth process fabricated ZnO nanostructures with flower-like morphologies through a hydrothermal process. For this ZnO nanorods synthesized in the initial step were treated with [Zn(OH)_4_]^2−^ solution. The obtained mixture was heated at 100 °C for 10 h in an autoclave. In this growth step, ZnO nanorods having diameters ranging from 250 to 500 nm and lengths from 3 to 5 μm rearrange themselves to form flower-like structures with about 5–7 μm radius. Figure 11a, b represents the FESEM images of flower-like ZnO microstructures and confirms the above said facts.

**Table 2:** ZnO nanostructures: morphology, methods of preparation, size, and other growth parameters

Morphology	Synthetic method	Growth reagents	Substrate	Anne. temp. (°C)	Anne. time (in h)	Diameter (nm)	Ref.
Microflowers	Hydrothermal method	Zn(NO_3_)_2_·6H_2_O, CTAB	–	–	–	5–7 μm	[51]
Nanoflowers	Hydrothermal method	Zn(NO_3_)_2_·6H_2_O, NaOH	–	400 600 800	1	Several μm	[52]
Quantum dot	Wet Chemical method	Zn(CH_3_COO)_2_·2H_2_O in DMSO, (CH_3_)_4_NOH·5H_2_O (TMAH)	Al_2_O_3_	–	–	2.5–4.5	[53]
Quantum dot	Sol–gel method	Zn(CH_3_COO)_2_·2H_2_O, Oleic acid	–	200 400 600 800	1	8	[54]
Quantum dots	Sol–gel method	Zn(CH_3_COO)_2_·2H_2_O, Tetraethylorthosilicate (TEOS)	Silica	200	1	5.7	[55]
				400		6.9	
				600		26.1	
				800		36.8	
Quantum dot	Sol–gel method	ZnCl_2_, SDS	–	200	3	4.5	[56]
				400		7.4	
				600		9.5	
				800		11.3	
Nanoparticles	Hydrothermal method	Zn(NO_3_)_2_·6H_2_O, trisodiumcitrate	–	–		100–150	[57]
Nanoparticles	Hydrothermal method	ZnCl_2_, SDS, ammonium hydrogen carbonate	–	200	3	5.5	[58]
				400		23.2	
				600		257	
				800		270	
Necked nanoparticle	Commercial	ZnO	–	400	–	20–150	[59]
Nanoparticles	Sol–gel method	(Zn(CH_3_COO)_2_·2H_2_O), Triton X-100	–	400	–	10	[60]
Nanoporous thin films	Electrodeposition method	ZnCl_2_	Ti	–	–	–	[61]
ZnO Coating	Atmospheric plasma-sprayed	Commercially available ZnO powders	Al_2_O_3_	–	–	10 µm	[62]
ZnO thin film	Sol–gel spin-coating method	Zn(CH_3_COO)_2_·2H_2_O, methanol	Glass	400 500 600 700	–	40–52	[63]
Hollow spheres	Template method	Zn(CH_3_COO)_2_·2H_2_O, dimethylformamide (DMF), glucose and absolute ethanol	Carbon microsphere	450	2	200–400 with shell thickness of 25 nm	[64]
Nanopyramids	Non-aqueous and surfactant free hydrothermal method	Zn(CH_3_COO)_2_·2H_2_O, benzylamine	–	–	–	Hexagonal base and height of ∼100 nm	[65]
Nanotetrapods	Vapor-phase growth	Zn foil	Al_2_O_3_	–	–	Tetrapods “legs” generally are 50–200 nm thick	[66]

From the literature review, it was observed that 1D ZnO nanostructures with few exceptions for their NO_2_ gas sensing properties operate at very high temperature starting about 200 °C which reduces their applicability at room temperature and also increases their practical cost for the gas sensing properties. Bai et al. [52] studied the growth of flower-like ZnO structures as a function of calcined temperature. ZnO nanorods formed were found to aggregate to larger extent with increase in annealing temperature so as to give a flower-like appearance. Morphology and aspect ratio of ZnO flowers ZnO nanostructures were also found to be depending upon the extent of annealing temperature.

### ZnO Quantum Dots and Nanoparticles

**Fig. 12:**
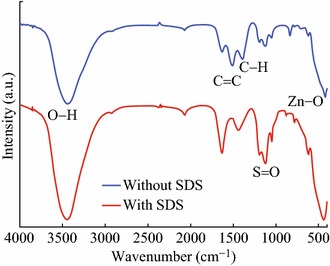
FT-IR spectra of the SDS-modified and unmodified quantum-sized ZnO nanoparticles. Reprinted with permission from [56]. © 2012 Elsevier B.V

Another approach to enhance the gas sensing properties of ZnO-based gas sensors is to reduce the particle size and to increase the surface-to-volume ratio. ZnO nanoparticles of only few nanometer crystallite size offer high density of grain boundaries and interfaces for the increased interaction of NO_2_ molecules for better and quick responses in electrical resistance. Quantum-sized ZnO nanoparticles have been reported in the literature through sol–gel methods using different capping agents so as to control their growth and size [54–56]. Forleo et al. [53] obtained quantum dots with mean crystallite size of the range 2.5–4.5 nm by a simple wet chemical method at room temperature. In a similar approach, Bai et al. [54, 55] reported that the size of the ZnO quantum dots can be controlled by the nature and concentration of the capping agents. They synthesized ZnO quantum dots at room temperature by a sol–gel process using oleic acid as capping agent. Oleic acid prevents the aggregation of clusters and slows the growth rate of ZnO crystal and orients the preferential growth along [0001] direction of ZnO crystal [54, 86]. Tetraethylorthosilicate (TEOS) is also found to perform similar functions as that of oleic acids [55]. The alkoxyl groups of TEOS form a capping layer of silica on the surface of ZnO which have been confirmed by the presence of absorption peak at 1,000 cm^−1^ with a small shoulder band at 890 cm^−1^[87, 88]. Sodium dodecyl sulfate (SDS) can also be used as surface acting agent for control the growth rate of ZnO nanoparticles [56, 58]. Surface modification of by SDS molecules on the surface of ZnO can be confirmed by FT-IR analysis. For SDS-modified ZnO quantum dots, a band near 1,200 cm^−1^ due to S=O stretching vibration of [SO_4_]^2−^ from the SDS molecule is observed [56, 89]. However, this band is missing in the FT-IR spectrum of non-modified ZnO nanoparticles (Fig. 12).

**Fig. 13:**
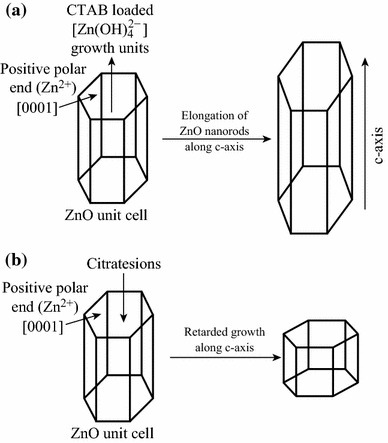
Comparison of growth mechanisms of ZnO nanorods in the presence of negatively charged surfactant (trisodiumcitrate) and positively charged (CTAB) surfactants

In another report, Rai et al. [57] synthesized ZnO nanoparticles from Zn(OH)_2_ precursor (prepared by co-precipitation method) using trisodiumcitrate, an anionic surfactant-assisted hydrothermal process. Due to the presence of negatively charged citrate ions, there is preferential absorption of these ions on the positively charged polar planes [0001] thus decreasing the growth rate along [0001] plane by competing with [Zn(OH)_4_]^2−^ growth units. As reported earlier in “1-D ZnO Nanomaterials (Nanorods, Nanowires, Nanotubes, and Nanofibres)” section, cationic surfactants like CTAB direct this growth unit along positive polar planes [0001] resulting in the elongation of ZnO nanorods [41]. Figure 13 represents a comparison for growth mechanism of ZnO in the presence of anionic (trisodiumcitrate) and cationic (CTAB) surfactants. In many other reports, nonionic surfactants such as Triton X-100 are also being used as capping agents to control the growth and morphology of ZnO nanostructures [60].

### ZnO Thin Films and Sheets

**Fig. 14:**
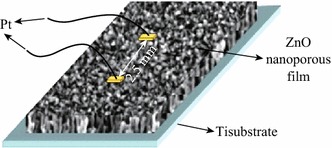
Schematic representation of the electrochemically deposited ZnO porous thin film on Ti substrate. Reprinted with permission from [61]. © 2012 Elsevier B.V

Two-dimensional nanostructure porous sensitive layer with comparatively larger surface-volume ratio and large number of reaction sites offers high and quick sensor response. The porosity of the surface of thin films directly influences the gas diffusion rate and chemisorption of oxygen and analyte gas so as to enhance the gas sensing properties [61]. Recently, Organic dye has been found to be useful for controlling the surface morphology and porosity of the ZnO thin films [61, 90–92]. The loaded dye can be removed from the deposited film by treating it with dilute base solution. A number of physical, chemical, and electrochemical methods for ZnO thin film deposition for NO_2_ gas sensing applications have been reported in the literature [61–63]. Bai et al. fabricated ZnO nanoporous thin films on Ti substrates through one-step electrodeposition method using three-electrode system constituting a reference saturated calomel electrode (SCE), Zn wire as a counter electrode, and working Ti electrode and eosin Y (EY) dye to control the morphology and porosity of the thin film [61] (Fig. 14).

**Fig. 15:**
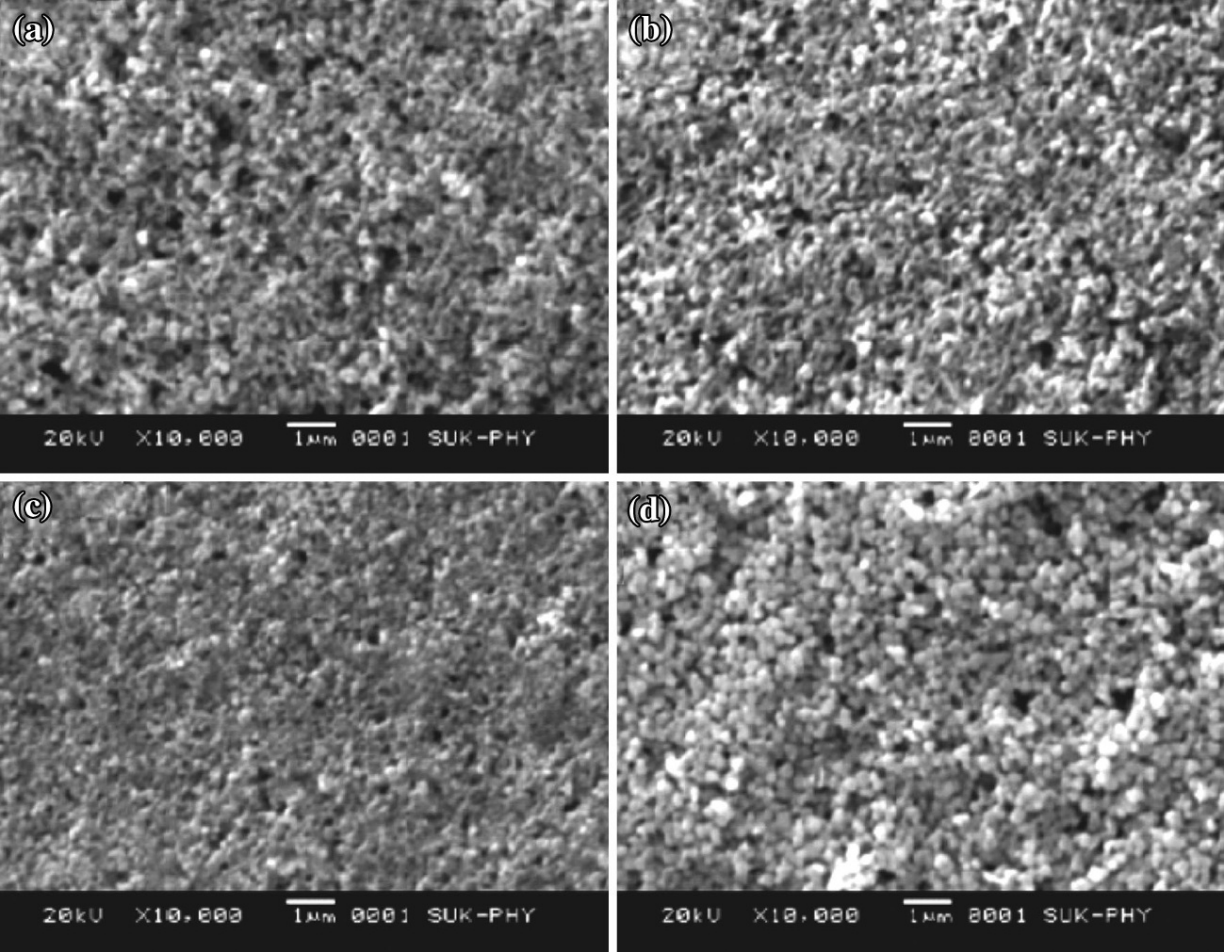
**a–d** SEM images of ZnO thin films annealed at 400, 500, 600, and 700 °C, respectively. Reprinted with permission from [63]. © 2011 Elsevier Ltd and Techna Group S.r.l

Zhang et al. [62] employed atmospheric plasma spray (APS) technique for depositing ZnO gas sensing surface on alumina plate having Au electrodes and a Pt heater on its two faces using commercially available ZnO powder. Annealing temperature has a marked effect on the crystallinity and crystallite size of the ZnO-based thin films [63]. Crystallite size was found to increase from 40 to 52 nm as annealing temperature was increased from 400 to 700 °C for ZnO thin films deposited using sol–gel spin-coating method on glass substrate by Chougule et al. [63] (Fig. 15).

The deposition of ZnO thin films physical methods, however, suffers from major drawbacks like high-cost fabrication techniques high energy working conditions [62].

### ZnO Materials with Other Morphologies

**Fig. 16:**
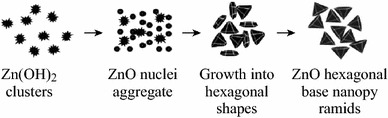
Growth process for the formation of ZnO nanopyramids by the solvothermal process. Reprinted with permission from [65]. © 2012 Elsevier B.V

ZnO nanostructures of various other types of morphologies including hollow spheres [64], nanopyramids [65], and nanotertrapods [66] have also been utilized for gas sensing applications (Table 2). As stated earlier, the porosity of the ZnO-based gas sensors influences the gas sensing properties, the ZnO hollow sphere can be used as effective NO_2_ gas sensors. Zhang et al. [64] for this approach synthesized ZnO hollow spheres templated by carbon microspheres. Ahmed et al. [65] proposed the formation of Zn(OH)_2_ precursor from benzylamine and zinc acetate for the formation of ZnO nanopyramids in non-aqueous medium. Figure 16 represents the growth process related to the formation of these pyramidal structures from Zn(OH)_2_ precursor.

ZnO tetrapods were grown by Calestani et al. [66] in a tubular furnace by thermal evaporation and controlled oxidation of Zn foil.

## Fabrication of ZnO-Based Gas Sensors for NO_2_

Depending upon literature reports, the fabrication of ZnO-based gas sensors for NO_2_ gas can be broadly classified into three categories.

### Direct Growth of ZnO Nanostructures on Substrate

The best studied sensors for NO_2_ gas are the porous ZnO-based nanostructured layers directly deposited through hydrothermal [29–32, 34, 48, 50], electrochemical deposition [33, 61], evaporation condensation [42–44], chemical bath deposition [38], electrospinning [45], sputtering [47], and atmospheric plasma spray [62] methods on the transducer surface such as alumina [31, 32, 47, 62], Si [33, 34], SiO_2_ [43, 45], glass [29, 30], quartz [38], etc. Among the various morphologies of ZnO nanostructures, 1-D nanostructures have attracted much attention recently as gas sensors because of their high surface-to-volume ratio. Fabrication of 1-D nanostructures and their potential use as gas sensors is a major concern of the nanotechnology now a day. A number of methods are reported in the literature for 1-D ZnO nanostructures utilized for gas sensing application. Out of these methods, simple hydrothermal method is reported for direct growth of 1-D ZnO nanostructures on various substrates [29–32, 34, 48, 50] (Table 1).

### Indirect Deposition of Grown ZnO Nanostructures on Substrate

Another approach for the fabrication of porous ZnO-based nanostructured gas sensors is pre-synthesis followed by coating of the as-synthesized ZnO nanomaterials of various morphologies such as 1-D nanorods, nanotubes, nanofibres, nanotubes, nanolines, nanowires [35–37, 39, 49], micro/nanoflowers [51, 52], quantum dots [53], nanoparticles [57, 59], hollow spheres [64], nanopyramids [65], and nanotetrapods [66] as thin/thick films/coatings [35–37, 39, 49, 51, 53, 57, 65, 66] on the transducer surface including alumina [35, 37, 39, 49, 51, 53, 57, 66] and SiO_2_ [65]. Doctor blade method has been reported for the coating of ZnO nanomaterials on the sensor substrate in the literature by Rai et al. [37, 51, 57].

**Fig. 17:**
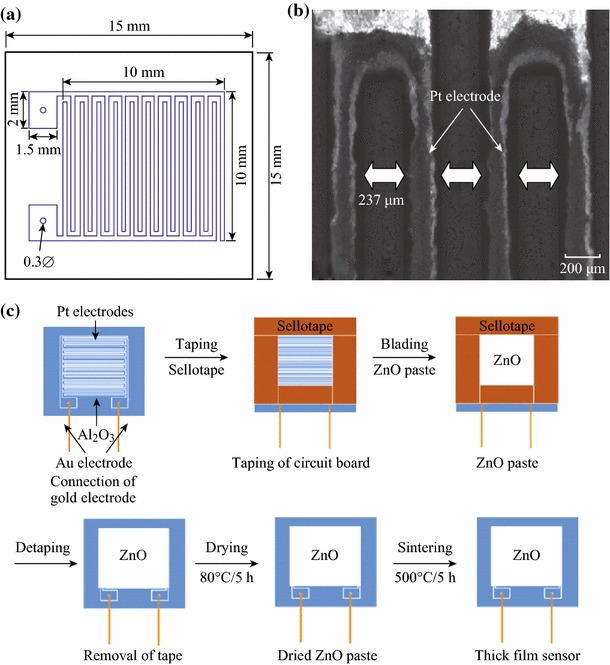
**a** Diagrammatic representation of alumina circuit board, **b** SEM image of alumina circuit board and **c** schematic illustration of doctor blading method coating ZnO sensor materials. Reprinted with permission from [37]. © 2012 Elsevier B.V

ZnO nanomaterials are mixed and grinded with α-terpinol as binder to form a paste which was coated by this method onto the cleaned alumina circuit board with cello tape on all the sides and having an interdigitated platinum electrodes to form films with thicknesses ranging from few hundred of nm to several hundred of microns. Schematic representation for the deposition of ZnO nanomaterials by doctor blade method is shown in Fig. 17. Coated thin films are annealed at high temperature for a definite time period. Au wire electrodes were connected with the help of Au or Ag paste for making sensor device [37, 51, 57]. Spin-coating technique is also reported in the literature for forming ZnO thin layers on SiO_2_ substrate by Ahmed et al. [65].

As ZnO nanostructured sensors exhibit maximum response at high temperature, a resistance heater of platinum is coated by screen printing on the backside of the substrate in direct growth as well as indirect deposition methods [31, 32, 35, 36, 62].

### Pelletization

**Fig. 18:**
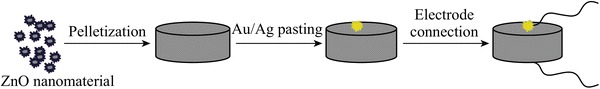
Schematic representation of the various steps involved for the fabrication of pelletized ZnO nanostructures for NO_2_ sensing

Another simple approach for the fabrication of ZnO-based nanomaterials as gas sensor for NO_2_ is the pressing of annealed ZnO nanomaterials into a pellet with different diameters and thicknesses under a high pressure of 7–10 MPa [40, 56, 58]. Before pressing the powders to a pellet form, suitable adhesion agent such as ethanol is mixed. Au wires are soldered on both sides of the pellet with the help of silver or gold paste to form a sensor element. The pellet-based sensor is subsequently dried and aged at high temperature to remove the adhesion agent and any adsorbates from its surface before applying for sensor application [40, 54–56, 58]. Figure 18 shows the steps for the fabrication of pelletized ZnO nanostructures for NO_2_ sensing.

## Factors Influencing Gas Sensing Properties

### Effect of Calcination Temperature and Size on Gas Sensing

As stated earlier, gas response increases and resistance decreases with decreasing the ZnO crystal size, and annealing temperature plays a significant role. For obtaining optimum gas sensing properties, ZnO nanostructured materials have to be calcined at appropriate temperature. It is well-known fact that, with increasing annealing or calcination temperature, the crystallinity of the ZnO nanomaterials is improved and the extent of stoichiometric defects in the form of oxygen vacancies is increased. Greater the number of such defects better is the gas sensing response. However, a very high annealing temperature causes the agglomeration of the particles, resulting in increased crystallite size and reduced specific surface which in turn retards the gas response [93, 94].

**Fig. 19:**
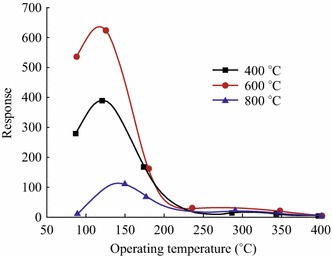
Sensor responses of ZnO nanorods calcined at 400, 600, and 800 °C temperatures. Reprinted with permission from [40]. © 2013 Elsevier B.V

Bai et al. [40, 52, 55] observed one such behavior for ZnO nanorods calcined at 400, 600, and 800 °C temperatures for 40 ppm NO_2_ gas at 120 °C operating temperature (Fig. 19). Maximum response was observed at 600 °C as compared to 400 and 800 °C annealing temperatures.

At low annealing temperature treatment, the ZnO nanomaterials have high-specific surface area due to small crystallite size but have poor crystallinity which is an essential requirement for the sensor applications. The value of annealing temperature at which maximum response is observed for NO_2_ gas further depends upon the morphology as well as on the method of synthesis of nanoparticles. Hjiri et al. [49] observed maximum response for ZnO nanofibres synthesized hydrothermally and subsequently annealed at 700 °C. ZnO quantum dots synthesized by Bai et al. [54, 55], Li et al. [56], and Fan et al. [58] annealed at 400 °C show a maximum response for 40 ppm NO_2_ gas.

### Effect of Operating Temperature and Concentration on Gas Sensing

**Fig. 20:**
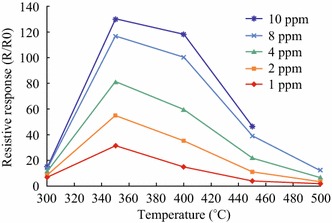
Gas sensing response of the ZnO-based sensor determined at different operative temperatures for different NO_2_ gas concentrations. Reprinted with permission from [35]. © 2013 American Chemical Society

From the literature study, it has been observed that there is a direct correlation between the concentration of analyte NO_2_ and operating temperature for ZnO-based gas sensors. As the species are chemisorbed on the surface of ZnO nanostructures, there is expected initial increase in the extent of adsorption due to the requirement of activation energy but at relatively high operating temperature desorption of the analyte gas occurs and the sensor response decreases [40, 41]. Moreover, with increasing operational temperature, the dominant process is the adsorption of O^2−^ which also lowers the ZnO sensor response. The sensor response indicated a linearly correlation with the concentration of NO_2_ at optimized value of the operative temperature [31, 32]. Ozturk et al. [29] observed that the change in the resistance increases with increasing concentration of NO_2_ gas from 100 ppb^−1^ ppm at high-temperature range of 150–200 °C for ZnO nanorods-based sensors. At low-temperature range, the recovery time of the sample is very large. Thus, under these conditions, ZnO-based sensors cannot be applied for gas sensing applications [29]. At high operating temperature, response time of the sensor is low. It is well known that ZnO is a well-known n-type semiconductor material and NO_2_ is an oxidizing gas which on the surface of ZnO nanorod surface gets reduced. This results in the increase in the resistance of ZnO nanorods which increases with NO_2_ concentration and subsequent adsorption. However, at very high NO_2_ concentration, surface reaction rate determines the gas sensor response due to the presence of insufficient adsorption sites [35, 95]. Figure 20 clearly demonstrates the variations of sensor response as a function of concentration of NO_2_ and operating temperature.

One of the major drawbacks of the reported ZnO-based gas sensors is the high operative temperature which is the main obstacle for their commercial applications. This drawback however can be removed or reduced to some extent by utilizing highly porous, with large specific surface area of ZnO nanosensors. Xu et al. [38] and Lu et al. [96] reported the photo-enhanced NO_2_ sensing applications of ZnO-ordered nanoclusters at room temperature under UV light illumination (Table 3). Photo-generated electrons present on the surface of the ZnO sensor under UV light illumination symbiotically reduce the adsorbed NO_2_ gas, enhancing the resistance and hence the sensor response [96].

**Table 3:** Comparison of performances of ZnO-based nanostructured NO_2_ gas sensors

ZnO morphology	Sensor assembly	Temperature (°C)	NO_2_ conc. (ppm)	*τ*_res_ (in seconds)	*τ*_rec_ (in seconds)	Gas response (*R*_g_/*R*_a_)	Ref.
Nanorods	Glass/seed layer/nanorods/Au	200	100^≠^	20	–	0.075^α^	[29]
	Glass/Au/seed layer/nanorods	200	1	84		0.3	
Nanorods	Glass/ITO/seed layer/nanorods/Ag	200	1	182	720	1.316^δ^ (in light)	[30]
				3–4	1,320	1.308^δ^ (in dark)	
Nanorods	Alumina/Pt/Zn thinfilm/nanorods	250	10^≠^	20 min	40–50 min	824^β^	[31]
Nanorods	Alumina/Pt/seed layer/nanorods	90	1	–	–	40.9^α^	[32]
		100				31.8^α^	
		110				11.2^α^	
Nanorods	Porous Si/Pt/nanorods/Pt	25–125	0.1–1	200–90	180–120	1.2–5.8	[33]
Nanorods	Si/Pt/nanorods	300	1	~9	~10	~100^α^	[34]
Nanorods Nanoprisms	Alumina/Au/nanomaterial	350	10	–	–	~130	[35]
Nanorods with nanovoids	Platinum interdigitated electrode	250	10	–	–	51.25	[36]
Au/ZnO nanorods	Alumina/Pt/nanorods	300	50	–	–	4.14	[37]
ZnO nanorods						10	
Hierarchical nanoclusters were built from 1D single crystal nanorods	Quartz/platinum/hierarchical nanoclusters	RT	1	–	–	1.6 (No UV) 4.1 (UV)	[38]
Ce/ZnO nanorods	Alumina/Pt/nanorods	100	2	390	–	75^α^	[39]
Needle-like nanorods	Au electrode pallet	120	40	–	–	624	[40]
Pencil-like nanorods		400				206	
Flower-like ZnO nanorods		400				44.8	
Pencil-like nanorods	Alumina tube with Au electrodes	400	40	34 (for 5 ppm)	80 (for 5 ppm)	206	[41]
Unbranched and branched* nanowires	Sapphire substrate/Au/nanowires	300	1 2 3 4 5	20, 17*	85, 65*	13.97^α^, 26.09*^α^	[42]
			2	30, 15*	100, 75*	18.74^α^, 30.51*^α^	
			3	55, 04*	120, 55*	27.49^α^, 53.85*^α^	
			4	35, 10*	100, 45*	36.71^α^, 77.79*^α^	
			5	40, 12*	100, 60*	43.43^α^, 106.27*^α^	
Nanowires	SiO_2_/Si/Ti/Pt/nanowires	225	5	44	5	~58	[43]
Functionalized nanowires	Alumina/Pt/nanowires	190	2	–	–	~0.5^α^	[44]
Nanobarbed fibers	SiO_2_/Si/nanobarbed fibers	210	0.03	90	36	1.58	[45]
Nanolines	SiO_2_/nanoline	–	–	–	–	–	[46]
Nanobelt	Sapphire substrate/nanobelt	350	10	120 (for 5 ppm)	150 (for 5 ppm)	1.81	[47]
Nanoneedle	Glass/nanobelt thin film	200	200	41	125	64^β^	[48]
Cacti-like structure				21	24	89^β^	
Nanoprisms	Alumina/Pt/nanoprism	350	1	–	–	~1.04	[49]
Nanofibers	Alumina/Pt/nanofibre					~1.25	
Nanotubes	Si/SiO_2_/nanotubes/Au	30	500^≠^	–	–	1.51	[50]
Microflowers	Alumina/Pt/microflowers	300	100			12.27	[51]
Nanoflowers	Pellet with Pt electrode	150	10	8	40	55	[52]
Quantum dot	Alumina/Pt/quantum dot	200	2	–	–	~1000^δ^	[53]
Quantum dot	Pellet with Ag electrode	290	5	7	35	280	[54]
Quantum dots	Pellet with Ag electrode	290	40	7 (for 5 ppm)	35 (for 5 ppm)	264	[55]
Quantum dot	Pellet with Au electrode	290	40	–	–	221.7	[56]
Nanoparticles	Alumina/Pt/nanoparticles	300	100	45	73	18	[57]
Nanoparticles	Pellet with Ag electrode	290	40	30	120	~230	[58]
Necked nanoparticle	Si/thin film paste/Au	200	0.2	13	10	100	[59]
Nanoparticles	Glass/thin film	200	100	6	17	~36.3^β^	[60]
Nanoporous thin films	Ti/thin film	100	40	–	–	544.8	[61]
ZnO Coating	Alumina/Au/thin film alumina/Pt/nanofibre	300	2.42	–	–	5.3 (in dry air) 30.8 (in wet air)	[62]
ZnO thin film	Glass substrate/thin film	200	100	6.72	52.62	37.2^β^	[63]
Hollow spheres	Alumina/hollow sphere/Au	240	10	31	–	140.6	[64]
			50	19		172.8	
			100	9		286.8	
Nanopyramids	SiO_2_/Au/nanopyramid	200	10	60	32	14.5	[65]
Nanotetrapods	Alumina/Au/nanotetrapod	300	20	–	–	20^α^	[66]

^*^Represents the parameters for branched nanowires

*α* = (*R*_g_ − *R*_a_)/*R*_a_, *β* = 100(*R*_g_ − *R*_a_)/*R*_a_, *δ* = (*I*_air_ − *I*_gas_)/*I*_gas_, *τ*_res_ = response time, *τ*_rec_ = recovery time, ≠ conc. in ppb

### Effect of Relative Humidity

**Fig. 21:**
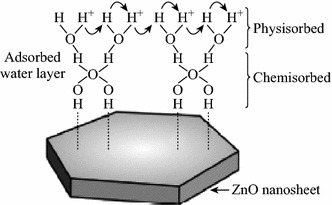
Physically and chemically adsorbed water molecules on the surface of ZnO nanosheets. Reprinted with permission from [104]. © 2013 Elsevier B.V

Another factor which affects the sensing performance of the ZnO nanostructure-based sensors is the environmental humidity. Physisorbed and chemisorbed water molecules are as shown in Fig. 21 form OH^−^ ions at above 200 °C which may remain attached to the ZnO sensor surface even up to 400 °C [97, 102–104]. Adsorbed water on the surface of ZnO lowers the gas response of due to reaction with surface oxygen thereby decreasing the baseline resistance and hence gas response [98, 99].

In relatively high humidity, adsorption of water molecules also prevents the chemisorption of oxygen on the surface of the sensor which is the prime requirement for the sensor response. This adsorbed water layer may act as a barrier between the ZnO sensor surface and the NO_2_ gas [97]. Deterioration of the gas response of ZnO gas sensors may further be increased due to the formation of chemisorbed OH^−^ from adsorbed water molecules on the surface [100, 101]. However, these ions can be desorbed from the surface by operating the sensor applications at high temperature [99]. In nutshell, the adsorption of water significantly lowers the gas response and repeatability of ZnO gas sensors. However, humidity can be removed from the sensor surface by high-temperature treatment. Table 3 clearly shows that maximum response is observed at temperature ≥400 °C for maximum reports irrespective to the morphology, size, and method of synthesis of ZnO and sensor fabrication technique.

## Conclusion

In summary, ZnO nanomaterials can be efficiently utilized as sensors for NO_2_ gas. ZnO nanorods and related 1-D materials, porous ZnO nanosheets and thin films with greater specific surface area, charge confinement ability and more reaction sites usually show better gas sensing properties than ZnO nanomaterials with other morphologies. Adsorption of the NO_2_ gas on the surface of the ZnO nanostructures is reduced by the transfer of the electrons from the conduction band which increases the resistance and increases the gas sensor response. The five parameters viz. gas response, recovery time, response time, selectivity, and detection limit depend upon the morphology, size, and surface area of the ZnO nanomaterials, the interaction between the gas and the sensor, concentration of NO_2_ gas, the operating temperature, etc. High-temperature annealing of the ZnO nanostructures results in the agglomeration into large entities. Due to this surface areas and hence gas sensing properties are reduced. It is mandatory to mention that 1D-ZnO nanomaterials provide a prospective base due to their crystallographic planes for their applications as durable conductometric gas sensors. Another factor which affects the sensing performance of the ZnO nanostructure-based sensors is the environmental humidity. Physisorbed and chemisorbed water molecules significantly lowers the gas response and repeatability of ZnO gas sensors. The major drawbacks and obstacle for the commercial applications of the reported ZnO-based gas sensors are the high operative temperature conditions and repeatability. The future research thus should focus to remove or reduce these limitations by synthesizing highly porous materials with large surface-to-volume ratio, optimization of the annealing and operating temperature, and using suitable additives like Pd, Pt, In, Cu, Nb, Mn, Ce, Si or other metal oxides so as to improve the gas response of ZnO-based nanosensors.
